# Mass Spectrometry-Based Discovery of *in vitro* Kinome Substrates

**DOI:** 10.5702/massspectrometry.A0082

**Published:** 2020-03-28

**Authors:** Naoyuki Sugiyama

**Affiliations:** 1 Graduate School of Pharmaceutical Sciences, Kyoto University, 46–29 Yoshida-shimoadachi-cho, Sakyo-ku, Kyoto 606–8501, Japan

**Keywords:** proteomics, LC-MS/MS, protein kinase, signal transduction

## Abstract

Protein phosphorylation mediated by protein kinases is one of the most significant posttranslational modifications in many biological events. The function and physiological substrates of specific protein kinases, which are highly associated with known signal transduction elements or therapeutic targets, have been extensively studied using various approaches; however, most protein kinases have not yet been characterized. In recent decades, many techniques have been developed for the identification of *in vitro* and physiological substrates of protein kinases. In this review, I summarize recent studies profiling the characteristics of kinases using mass spectrometry-based proteomics, focusing on the large-scale identification of *in vitro* substrates of the human kinome using a quantitative phosphoproteomics approach.

## INTRODUCTION

Reversible protein phosphorylation mediated by protein kinases and phosphatases is one of the most frequent posttranslational modifications and plays significant roles in a variety of biological events, especially in eukaryotic cells. Protein phosphorylation regulates various protein functions, including enzymatic activity, cellular localization and protein–protein interactions. Furthermore, protein kinases themselves are regulated by phosphorylation by other kinases or autophosphorylation.^1)^ Kinase-mediated protein phosphorylation is one of the main components of cellular signal transduction that broadly regulates cellular functions, and includes cell growth, division, apoptosis, and mitosis. The dysregulation of signal transduction caused by the overexpression or abnormal activation of a protein kinase is closely related to various diseases, including cancer.^2,3)^ Small molecular weight kinase inhibitors and antibodies have been developed and are now approved for use as molecular-targeting agents in cancer therapy.

Human genome sequencing analyses have revealed at least 518 genes that encode protein kinases,^4)^ and approximately 70% of all human proteins contain at least one phosphorylation site.^5)^ This means that each protein kinases are involved in literally hundreds of phosphorylation events assuming that all kinases have an equal number of substrates, and the resulting phosphorylation networks are quite complicated. Although a large body of information is available only for a few kinases that have been well studied, easily assayed, and/or genetically associated with a disease,^6)^ the functions and physiological substrates of hundreds of other kinases remain unclear. Therefore, it is assumed that the currently known signaling pathways are not sufficient to explain all of the cellular events. To understand the overall picture of intracellular signal transduction networks based on protein phosphorylation, a complete set of kinase–substrate relationships (KSRs) of all protein kinases is required. Actually it is essential that the fluctuation of all expressed protein kinases (kinome) and their substrates (phosphoproteome) are analyzed comprehensively, and additionally a research strategy focused on revealing the KSRs based on kinome and phosphoproteome is also required, as discussed below.

Phosphoproteomics based on liquid chromatography-mass spectrometry (LC-MS) and the highly selective enrichment of phosphopeptides enables us to identify protein phosphorylation without a bias, although the observability of the phosphosites depends on the amount of the phosphorylated proteins and sites. From recent advances in phosphoproteomics, more than 10,000 phosphorylated sites were identified in a single LC-MS experiment.^7)^ Using phosphoproteomics, in combination with quantitative analysis using stable isotope labeling, the dynamics of phosphorylation of intracellular proteins can be comprehensively observed. Quantitative phosphoproteomics has been utilized to reveal several signal transduction mechanisms such as EGF signaling,^8)^ mitosis,^9)^ and cell differentiation.^10)^ Large-scale phosphoproteomics has become a very powerful tool for revealing the entire picture of signal transduction mechanisms; however, estimating the activity of each kinase *in vivo* from a phosphoproteome dataset continues to be a challenge because many kinases function simultaneously. In a comprehensive phosphoproteome study, less than 40% of human phosphoproteins can be mapped to the KEGG^11)^ pathway database at the protein level,^12)^ and more than 95% of reported phosphosites have no known responsible kinase or biological function.^13)^

Computational tools for predicting the responsible kinase or potential substrates, including KinasePhos,^14)^ NetPhorest^15)^ and Networkin,^16)^ have been developed and have been summarized in a recent review.^17)^ Enrichment analyses using KSEA^18)^ and PTM-SEA^19)^ are based on known KSRs and have also been applied to estimate kinase activities from quantitative phosphoproteome datasets.^20)^ Most of the tools for making such predictions utilize substrate sequence models of protein kinases, which are obtained by experiments and/or public databases, such as PhosphoELM^21)^ and PhosphoSitePlus,^22)^ as training datasets, and in some cases integrate other information, such as protein–protein interactions, subcellular localization and time-course phosphoproteome datasets. Common constraints of these tools are the limited coverage of the kinome and a lack sufficient information concerning KSRs of little-studied protein kinases.

In this review, approaches to the characterization of protein kinases and their applications are discussed in an attempt to reveal comprehensive KSRs, focusing on mass spectrometry-based approaches for the identification of *in vitro* kinase substrates.

## COMPARISON OF METHODS FOR PROFILING KINASE SUBSTRATE SPECIFICITIES

**Figure 1:**
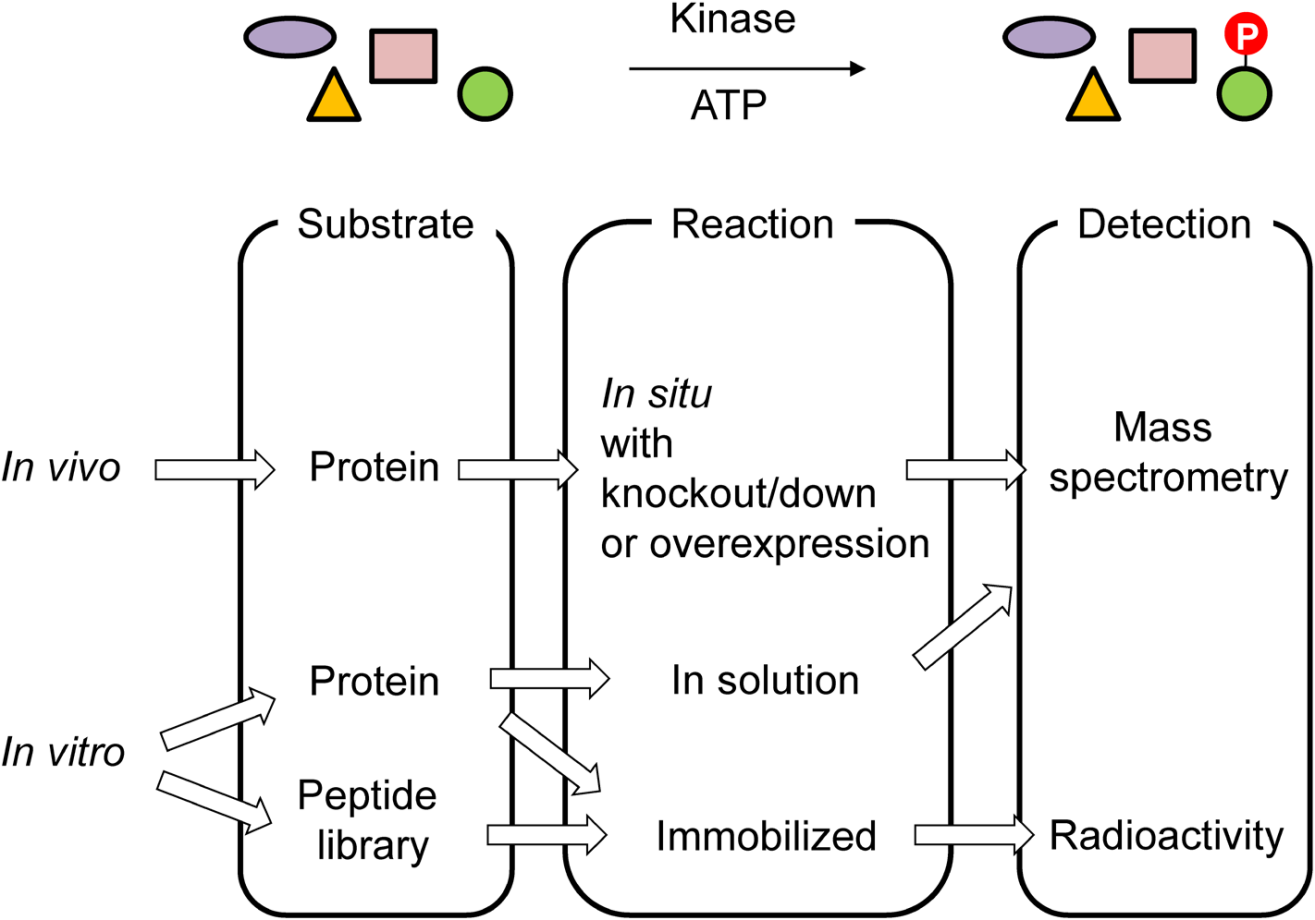
Fig. 1. Approaches for the identification of kinase substrates.

To date, many approaches for the profiling of kinase substrate specificities have been developed. These approaches, which are summarized in Fig. 1, can be mainly separated into two groups: namely, *in vitro* and *in vivo* kinase profiling. In the case of *in vivo* kinase profiling, the overexpression or knockout/knockdown of a specific kinase^23)^ and treatment with an activator^24)^ or a kinase inhibitor with a known specificity^25–28)^ are utilized as a perturbation. After the perturbation, changes in the phosphoproteome are measured by using a phosphoproteomics approach. Proteins and phosphorylated sites in which phosphorylation levels fluctuate with perturbations are considered as possible substrates of a target kinase. *In vivo* kinase profiling can provide physiologically relevant information concerning a kinase substrate, although *in vivo* kinase profiling is generally more laborious than *in vitro* studies, and it is difficult to distinguish true substrates that are directly phosphorylated by target kinases from indirect secondary reactions by downstream kinases. To avoid secondary reactions, the combined use of an analog-sensitive mutant strain and compounds that do not inhibit any wild-type kinases but inhibit analog-sensitive kinases was applied to *in vivo* kinase profiling,^23,29)^ as discussed below.

Typical combinations of substrate sources, reaction conditions and detection methods are shown here.

In *in vitro* kinase profiling, an individual protein kinase, which is usually prepared as a recombinant protein, is reacted with a substrate source. The main advantage of *in vitro* kinase profiling is that direct substrates are easily identified due to their simple reaction system. Even when a cell lysate is used as a substrate source, secondary reactions rarely occur due to the relatively low concentration of endogenous kinases.^12)^ However, the loss of the subcellular localization and kinase concentration, which do not reflect intercellular conditions, may result in the identification of nonphysiologically relevant kinase substrate pairs. As a substrate source, a synthetic peptide library and cell lysate are allowed to react with the target protein kinase in an *in vitro* assay. When an immobilized peptide library is applied to kinase profiling, various detection systems for phosphorylation are feasible since there is no need to sequence the immobilized peptides. Typically, the incorporation of radioisotope-labeled phosphate using γ-^32^P-ATP is utilized due to its high sensitivity.

## IMMOBILIZED PEPTIDE LIBRARIES

Peptide and protein arrays are known to be a very high-throughput strategy for profiling protein functions by monitoring protein–protein interactions or enzymatic reactions. For profiling kinase substrate preferences, a randomized peptide library immobilized on beads^30)^ or arrays,^31,32)^ natural protein-derived peptides^33)^ and protein arrays^34)^ have been utilized.

A positional scanning peptide library^31,32)^ is one such randomized peptide-based approach. In this technique, degenerated peptides, in which the phosphorylated acceptor and another position are occupied by a specific amino acid, are immobilized at each spot. The detected phosphorylated spots represent the importance of the specific positions and amino acids for the preference of a target kinase for the substrate and are easily converted into a substrate model, such as a position-specific scoring matrix.

Using a positional scanning peptide library, a kinome-wide profiling yeast protein kinases was carried out.^32)^ Phosphorylation motifs were identified for 61 out of the 122 tested yeast kinases. This large-scale yeast kinome profiling data enabled the clustering of yeast kinases based on phosphosite specificity, and the obtained phosphorylation motifs were consistent with known *in vivo* substrates of some kinases. A kinome-wide profiling of human protein kinases using a protein array was reported by Newman *et al*.^35)^ They profiled *in vitro* substrates from 289 human protein kinases using a protein-immobilized microarray and obtained 24,046 KSRs and 300 phosphorylation motifs. They also constructed KSR networks that were thought to be physiologically relevant by integrating the *in vivo* phosphoproteome data acquired by MS-based phosphoproteomics and known KSRs from the literature and public databases. Finally, phosphosite-level KSR networks connecting 230 kinases to 2,591 phosphorylation sites in 652 substrate proteins were constructed, although the phosphosites were not directly identified by the *in vitro* study.

## IDENTIFICATION OF *IN VITRO* KINASE SUBSTRATES USING MASS SPECTROMETRY

The mass spectrometry-based *in vitro* profiling of kinase substrates using biological samples was first reported by Knebel *et al.*^36)^; since then, a number of methods have been developed, as reviewed elsewhere.^37)^ In a typical workflow, crude mixtures of proteins extracted from cells (or their digested peptides) are reacted with a recombinant protein kinase. The reaction mixture is digested with a specific proteinase or peptidase. Finally, the phosphopeptides are enriched and then measured by LC-MS/MS. The main advantages of mass spectrometry-based approaches are the large-scale identification of phosphorylated peptides and obtaining direct evidence of phosphosite localization using MS/MS spectra. Unlike a position scanning peptide library, the identification of *in vitro* substrates provides richer information, such as the cooccurrence frequency of specific amino acid combinations in multiple positions in the immediate vicinity of the phosphosites. Furthermore, a portion of the identified *in vitro* substrates can be identified as physiologically significant substrates by using a biological sample as a substrate source.

The *in vitro* kinase assay was applied to the profiling several to tens of protein kinases by using a cell lysate,^38)^ digested protein^39,40)^ and a human peptide library expressed in *E. coli*^41)^ as a substrate source. Though the number of identified substrates depends on the kinase activity, specificity and analysis platform, hundreds to thousands of *in vitro* substrates of each kinase were identified.

As mentioned above, the main disadvantage of the *in vitro* kinase assay compared to *in vivo* profiling is the low overlap of the *in vitro* kinase substrate relationships with the physiologically observed ones. To identify more physiologically relevant substrates, combinations of an *in vitro* assay and *in vivo* experiments have been developed.^42–44)^ In addition, purification of the kinase–substrate complex^45)^ and the photocrosslinking of the kinase and substrate using ATP analog^46)^ also enables the identification of more significant substrates by focusing on the interacting substrate proteins.

## ATP ANALOG-SENSITIVE KINASES

Protein kinases catalyze the transfer of the γ-phosphate group from ATP to the hydroxy group of serine, threonine and tyrosine (also histidine, aspartic acid, lysine and arginine in prokaryotes) residues of their substrates. A mutation from bulky amino acids to smaller amino acids in a gatekeeper region increases the space of the ATP binding pocket. This technique can be used in two different ways, namely, the selective activation of a mutant kinase using an ATP analog as a phosphate source and the selective inhibition of a mutant kinase using a bulky ATP-competitive small molecule (reviewed in^47)^). The former and latter were mainly applied in *in vitro* and *in vivo* kinase profiling experiments, respectively. In both cases, the effects of kinases other than a target analog-sensitive mutant are reduced when this technique is used. The introduction of a chemical tag using γ-thiophosphate derivatives of the ATP analog was also utilized to enrich substrate proteins of a target analog-sensitive mutant.^48–50)^

ATP analog-sensitive kinase-based approaches are very powerful tools for reducing the effects of other endogenous kinases and accurately identifying positive kinase substrates in both cases; however, this strategy is applicable only if analog-sensitive kinases are available. Furthermore, wild-type kinases may also utilize the ATP analog as a phosphate source to some extent.^51)^

## LARGE-SCALE *IN VITRO* KINOME PROFILING USING PHOSPHOPROTEOMICS APPROACHES

Mass spectrometry-based *in vitro* kinase assays enable the large-scale identification of *in vitro* kinase substrates; however, the number of protein kinases employed in an individual study is limited to several dozen. We performed an *in vitro* kinome-wide profiling of human protein kinases, combined with a highly selective method for the enrichment of phosphopeptides. Details of the studies are described in the following sections.

### Phosphopeptide enrichment

In general, phosphorylated peptides show a lower sensitivity compared to nonphosphorylated peptides in an LC-MS/MS analysis in the positive ion mode. Furthermore, the stoichiometric ratios of phosphoproteins in cells are dramatically lower than those of nonphosphoproteins. In an LC-MS/MS analysis with data-dependent acquisition, only a few phosphopeptides can be identified without an enrichment or a fractionation step. Therefore, the selective enrichment of phosphoproteins or phosphopeptides is essential to effectively identify the phosphoproteome. Immobilized metal ion chromatography (IMAC)^52,53)^ and metal oxide chromatography^54–56)^ have frequently been utilized for the enrichment of phosphopeptides in phosphoproteome analyses due to their low cost and compatibility with LC-MS. The major drawback of both approaches is the low selectivity for phosphopeptides, *i.e.*, acidic peptides that contain multiple aspartic acids and/or glutamic acid can also bind to both of the materials. To overcome the low selectivity, several modified approaches have been developed (reviewed in^57)^). Converting the carboxy groups of aspartic acids and glutamic acids in peptides to methyl esters^58,59)^ and optimization of the pH and solvent^60)^ reduce the adsorption of acidic peptides to IMAC beads. Enhancer-mediated enrichment also improves the selectivity for phosphopeptides. Metal oxide chromatography using acidic amino acids,^61)^ 1-octanesulfonic acid^62)^ and aromatic carboxylic acids^55)^ has also been reported.

**Figure 2:**
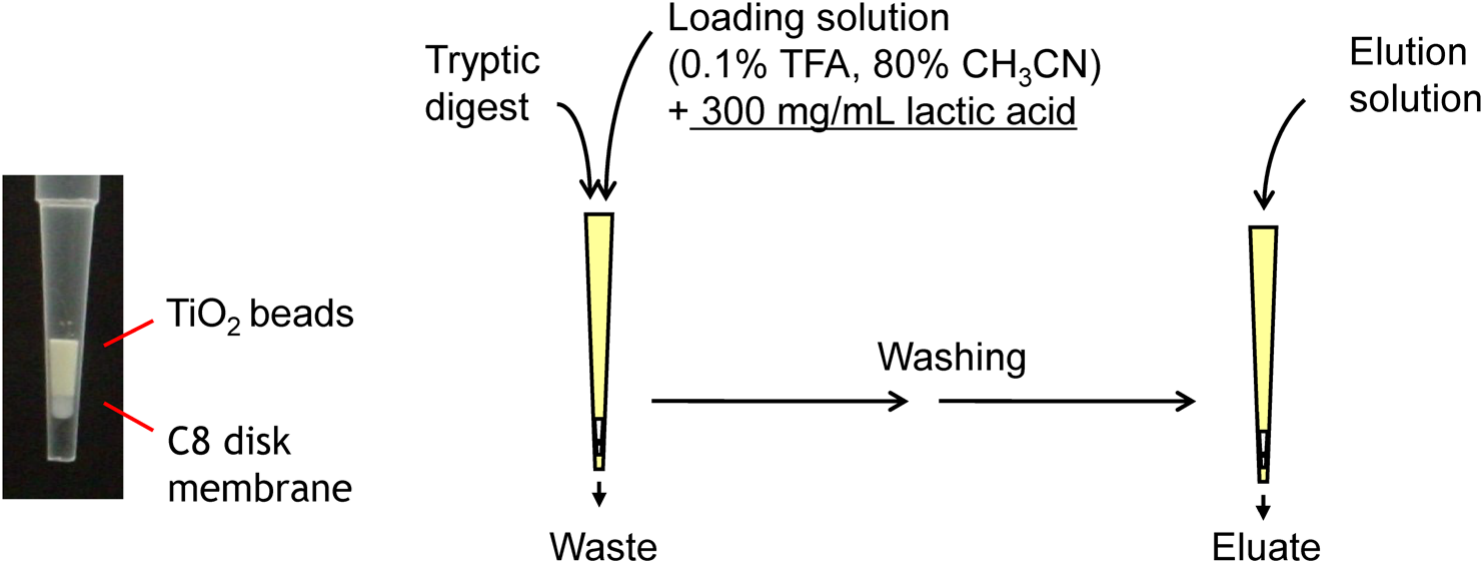
Fig. 2. Enrichment of phosphopeptides using hydroxy acid-modified metal oxide chromatography (HAMMOC).

To perform high-throughput and large-scale phosphoproteomics, we developed a method for the highly selective enrichment of phosphopeptides using titania (TiO_2_) and zirconia (ZrO_2_) with aliphatic hydroxy acids, which is referred to as hydroxy acid-modified metal oxide chromatography (HAMMOC)^56)^ (Fig. 2). In this approach, aliphatic hydroxy acids, including lactic acid, were dissolved in a solvent for metal oxide affinity chromatography. A high concentration of hydroxyl acid, which prevents the binding of acidic peptides to metal oxide beads, provided an extremely high selectivity for phosphopeptides. Furthermore, aliphatic hydroxy acids could be easily removed after enrichment by desalting using a reverse-phase column due to their hydrophilicity. Therefore, the HAMMOC method is highly compatible with LC-MS analysis. We compared the enhancement effects of aliphatic acids and previously reported additives described above for the enrichment of phosphopeptides. Lactic acid and β-hydroxy propionic acid were found to be the best enhancers for titania- and zirconia-metal oxide chromatography, respectively. The high selectivity of the HAMMOC approach enabled a large-scale identification of phosphopeptides from complex samples without the need for any prefractionation methods.

A metal oxide chromatography tip is prepared by packing bulk-titania beads into a C8-membrane StageTip.^25)^ Tryptic digests of the cell lysate are loaded onto the tip in the presence of a 300 mg/mL lactic acid. After washing twice, phosphopeptides are eluted using an aqueous base solution such as 0.5% piperidine. The fractionation with a successive elution improved the number of identified phosphopeptides in the following LC-MS/MS analysis.^76,77)^

### Large-scale identification of *in vitro* substrates of Erk1, PKA, and AKT1

A HeLa cell lysate was dephosphorylated by treatment with a thermosensitive alkaline phosphatase, and the added phosphatase and endogenous protein kinases and phosphatases were then heat denatured. Recombinant human protein kinases were individually added to the lysate and allowed to react in the presence of ATP, metal ions and some additives. After the reaction and subsequent reductive alkylation, the mixture was digested with a Lys-C endopeptidase and trypsin. Phosphopeptides were enriched using the titania-HAMMOC method and then analyzed by nanoLC-MS/MS.

This method was first applied to profiling three types of human recombinant protein kinases, and 3,585, 4,347, and 1,778 *in vitro* substrate sites for PKA, ERK1, and AKT1, respectively, were identified.^63)^ Most of the phosphorylation motif sequences extracted from the *in vitro* substrates were in agreement with known motif sequences; however, unreported motifs that reflect the specificity of each kinase in detail were also obtained. This result suggests that the larger number of kinase substrates enabled us to more accurately construct a substrate model that describes the substrate preferences of a kinase.

### Kinome-wide profiling

**Figure 3:**
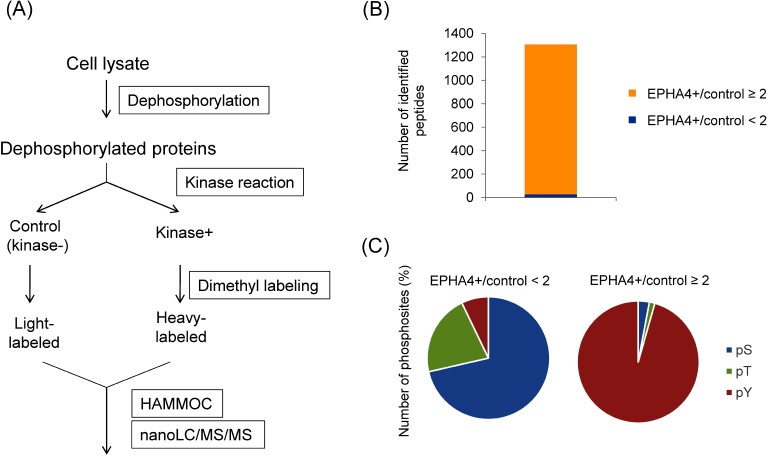
Fig. 3. Quantitative *in vitro* kinome profiling.

The method for the identification of *in vitro* kinase substrates was extended to kinome-wide profiling.^12)^ In a pilot study, we found that a few endogenous phosphosites were not dephosphorylated by a phosphatase treatment. The remaining phosphoproteins in the substrate source may result in the identification of false-positive substrates. To discriminate true substrates from inherent phosphosites, a stable isotope-based quantitation approach was used through kinome-wide profiling (Fig. 3A). Equal amounts of dephosphorylated lysates were reacted with or without a kinase and dimethylated using isotopically labeled formaldehyde. The use of a criterion that at a ratio of kinase-treated to mock control, permitted unreliable substrates derived from inherent phosphosites to be rejected (Figs. 3B and 3C). We investigated 354 wild-type and 21 mutant human recombinant protein kinases and 10 lipid kinases using a quantitative approach. As a result, 175,574 *in vitro* KSRs were identified, and a total of 1,576 phosphorylation motifs targeted by 303 kinases were obtained. To the best of our knowledge, this is currently the largest list of kinase–substrate and motif–kinase relationships that is currently available.

(A) Workflow of quantitative *in vitro* kinome profiling. Dephosphorylated cell lysate samples were individually reacted with 375 recombinant human protein kinases. Control (not treated with kinase) and kinase-treated samples were isotopically labeled and then analyzed by LC-MS/MS. Peak area ratios of kinase-treated (heavy-labeled) to controls (light-labeled) in all of the identified phosphopeptides were quantified by nanoLC-MS/MS. (B) Distribution of the peak area ratio in an *in vitro* assay with tyrosine kinase EPHA4. The number of identified monophosphorylated peptides with peak area ratios (EPHA+/control) greater than or equal to 2 and less than 2 are shown highlighted in yellow and blue, respectively. (C) The number of phosphorylated serine, threonine and tyrosine residues obtained by the *in vitro* reaction with EPHA4. Most of the identified phosphosites with a peak area ratio ≥2 were phosphotyrosines, which reflect the substrate preference of the tyrosine kinase EPHA4. In contrast, phosphosites showing a peak area ratio <2 were derived from inherent phosphopeptides that were not dephosphorylated by the phosphatase treatment.

Based on *in vitro* kinase substrates, serine threonine kinases were classified by a clustering analysis. The classification pattern in our study was generally similar to the kinome phylogenic tree reported by Manning *et al.*,^4)^ except that our classification strongly reflected a phosphoacceptor preference.

## PHOSPHOACCEPTOR PREFERENCES OF THE KINOME

Protein kinases have a preference for phosphoacceptors, namely, serine/threonine kinases (STKs) and tyrosine kinases (TKs) specifically phosphorylate Ser/Thr and Tyr, respectively, and most STKs show a Ser preference rather than a Thr preference. The common structure of protein kinases has been extensively investigated based on X-ray crystallography. All known protein kinases have a kinase domain, which is typically approximately 300 amino acids in length and largely consists of a β-stranded N-terminal lobe, an α-helical C-terminal lobe and a hinge region connecting both lobes (reviewed in^1,64)^). The activity of a protein kinase is regulated by the phosphorylation of a specific residue in the activation loop located in the C-terminal lobe. There are some reports describing correlations between the phosphoacceptor preference and the amino acid sequence in the kinase domain.^65–67)^ For example, Chen *et al.* reported that the amino acids following the conserved motif “DFG” at the N-terminus of the kinase activation loop, denoted as DFG+1, were strongly correlated with a Ser/Thr preference in the phosphoacceptor, and a mutation at this site changed the S/T preference of some kinases.^65)^ Using a filter binding assay, they analyzed the *in vitro* substrate preference and found that Ser-directed kinases have a larger number of hydrophobic residues (Leu, Phe, and Met) at the DFG+1 position, whereas Thr-directed kinases contain branched aliphatic residues (Ile and Val) at this site. Our dataset of *in vitro* kinome profiling are consistent with a part of the report.^12)^ Kinases with Phe and Val at the DFG+1 position exclusively showed serine- and threonine-directed activity, respectively. Kinases with Met at this position show a slightly weaker preference than those with Phe. In contrast to the report by Chen *et al.*, the phosphoacceptor preference of kinases with Ser at DFG+1 was not exclusive to Ser, and there was no stringent rule for kinases with other amino acids at this position.

Other positions, including APE-4 located in the activation segment and HRD+2 in a catalytic loop, also affect phosphoacceptor preference.^66)^ Nek10, which belongs to the NimA-related kinases (Neks), showed dual specific kinase activity, unlike other Nek kinases. This kinase contains an isoleucine, which is typically found in a tyrosine kinase, at the APE-4 position and a threonine, which is uncommon in both serine/threonine and tyrosine kinases, at the HRD+2 position. Mutations at these positions in Nek10 and other Nek kinases switched the substrate preferences.

In addition to phosphoacceptor preference, certain amino acids in the kinase domain are highly correlated with substrate specificity^67,68)^ and regulatory functions.^69)^ A computational tool was developed to predict which residues within the kinase domain contribute to substrate specificity based on a position-specific scoring matrix obtained by a position scanning peptide library.^68)^

## PERSPECTIVE

Proteomic approaches for profiling kinases have provided a great deal of information concerning substrate preferences, functions and regulatory mechanisms. The identification of true physiological substrates remains a major problem since substrate models of kinases, such as phosphorylation motifs, are not stringent, and the same motifs are shared by multiple kinases. According to our dataset, most of the consensus sequences of the tested protein kinases contain only 1 or 2 fixed amino acids, and not more than 3 residues, other than phosphoacceptors. Of course, other motif styles, such as the position weight matrix, contain much more information; however, completely distinguishing the substrate selectivity of kinases continues to be a difficult task.

Among the phosphorylated sites obtained by the *in vitro* profiling of 375 protein kinases in our study, only 24% overlap with the deep phosphoproteome dataset^5)^ of HeLa cells. Since the substrate specificities of most kinases are influenced by multiple factors, such as cellular colocalization and scaffold proteins,^70)^ primary structure-based substrate models are not sufficient to permit the substrate preferences of kinases to be described. However, *in vitro* kinase assays have enabled the discovery of novel kinase–substrate pairs, when carried out in combination with other *in vivo* experiments.^71,72)^ The results suggest that the *in vitro* kinase profiling dataset contains physiologically relevant substrate information to some extent, and the possibility exists that the dataset can be extrapolated to *in vivo* experimental phosphoproteome data. The combined use of other proteome-widescale datasets, including protein–protein interactions^73)^ and subcellular localizations,^74,75)^ will also be helpful in estimating physiologically relevant KSRs. Large-scale datasets of *in vitro* KSRs obtained by mass spectrometry-based technologies are expected to facilitate the elucidation of whole signal transduction networks and the development of more reliable computational tools, making it possible to find new rules regarding the substrate preferences of a kinase and to design artificial substrate peptides with higher specificies and sensitivities.

## Notes

Mass Spectrom (Tokyo) 2020; 9(1): A0082
